# Determinants of teenage pregnancy in Malawi: a community-based case-control study

**DOI:** 10.1186/s12905-024-03166-0

**Published:** 2024-06-08

**Authors:** James John Kalulu, Jeremot Masoambeta, William Stones

**Affiliations:** 1 Department of Health Systems and Policy, School of Global and Public Health, Kamuzu University of Health Sciences, Private Bag 360, Blantyre 3, Chichiri, Malawi; 2 University of Malawi Medical Scheme, P. O. Box 278, Zomba, Malawi; 3 National Statistics Office, P. O. Box 333, Zomba, Malawi; 4 Department of Public Health and Obstetrics & Gynecology, Centre for Reproductive Health, Kamuzu University of Health Sciences, Private Bag 360, Blantyre 3, Chichiri, Malawi

**Keywords:** Teenage pregnancy, Adolescent pregnancy, Malawi.

## Abstract

**Background:**

Teenage pregnancies are a global concern. Malawi is one of the countries with the highest teenage pregnancy rates despite government efforts to reverse the situation and yet studies on determinants of teenage pregnancy are rare with some factors remaining unexplored. Therefore, this study aimed to identify factors associated with teenage pregnancies in Malawi.

**Methods:**

This was a community-based case-control study that used secondary data from the 2015-16 Malawi Demographic and Health Survey from all 28 districts of Malawi. The study population comprised women aged 20–24 who participated in the survey. The study ran from September 2021 to October 2022 and used a sample size of 3,435 participants who were all women aged 20–24 in the dataset who met the inclusion criteria. Data were analysed using Stata 16 software. Logistic regression analyses were used to determine factors. Variables with a P value of < 0.1 in the univariable analysis were included in the multivariable analyses, where statistical significance was obtained at a P value < 0. 05.

**Results:**

Data on 3435 participants were analysed. In multivariable analyses: no teenage marriage (AOR 0.13); secondary education (AOR 0.26); higher education (AOR 0.39); richest category of wealth index (AOR 0.51), use of contraception (AOR 3.08), domestic violence by father or mother (AOR 0.37) were found to be significant factors.

**Conclusion:**

This study identified determinants of teenage pregnancy. The government has to sustain and expand initiatives that increase protection from teenage pregnancy, reinforce the implementation of amended marriage legislation, introduce policies to improve the socioeconomic status of vulnerable girls and increase contraceptive use among adolescent girls before their first pregnancy. Further research is also recommended to resolve inconclusive results.

## Background

Teenage or adolescent pregnancy occurs in a woman aged 10 to 19 years [1]. They are a global concern [2] and account for 11% of all deliveries worldwide and 23% of the disease burden in Disability Adjusted Life Years (DALYs) from pregnancy and childbirth among women of all ages [3]. Adolescent pregnancies are more likely to occur in marginalized communities [2], with nearly 95% occurring in developing countries [4]. Although there has been a global decline in trends of adolescent pregnancy for the past two decades, adolescent fertility has remained high in sub-Saharan Africa, at 101 births per 1,000 adolescent women [5].

Teenage pregnancy is both a medical and a public health concern due to its detrimental effects on the mother, baby and society. Its significant impact is felt on adolescents’ health and socioeconomic lives, especially girls [6–8]. Regarding health consequences, studies have shown a higher risk of adverse pregnancy outcomes among adolescents than among non-adolescents [9]. These negative outcomes include pregnancy and childbirth complications such as eclampsia, haemorrhage, systemic infections and unsafe abortions that contribute to maternal morbidity and mortality and lasting health problems [9, 10]. Babies born to mothers under 20 years of age face higher risks of low birth weight, preterm delivery and severe neonatal conditions [2, 9]. Social consequences for unmarried pregnant adolescents may include stigma, rejection, and violence by partners, parents and peers [7]. Teenage pregnancy and childbearing often lead to early school dropout, jeopardising girls’ future education and employment opportunities [7, 8]. The lower age of first childbearing is a determinant of higher fertility, contributing to high population growth [11, 12]. The growth and development of children born to teenage girls are likely to be affected by the socioeconomic status of their mothers [12].

Despite several detrimental effects to the mother, baby and society that come from teenage pregnancy [6–8], Malawi has been known to be among the top countries with a higher proportion of teenage pregnancies regionally and globally [6, 13, 14]. Currently, adolescent childbearing in Malawi is at 29%, a slight increase from a declining trend of 35% in 1992 to 26% in 2010 [15].

Concerted efforts to curb teenage pregnancy in Malawi date back to 1994 with the adoption of the International Conference for Population and Development (ICPD) Program of Action [16] and later on the introduction of the Youth Friendly Health Services (YFHS) program in 2007 [17]. The YFHS program addresses adolescent pregnancy through the ‘minimum package for YFHS’, which includes health promotion and counselling, delivery of health services, referral and follow-up [17]. Despite these efforts, Malawi continues to register an undesirably higher number of teenage pregnancies, as evidenced in the three preceding Malawi Demographic and Health Surveys (MDHS) of 2005, 2010 and 2015 [15].

Globally and regionally, some of the well-known determinants of teenage pregnancy include early sexual activity, marriage, low level of education, low socioeconomic status, lack of knowledge of reproductive health, low contraceptive use, family disruption and lack of power to negotiate for safer sex [12, 18–21]. In addition, context-specific factors such as quality of health services, place of residence, domestic violence and ethnicity have influenced adolescent pregnancy [19, 21, 22].

In Malawi, studies on determinants of teenage pregnancy are rare. Previous studies mainly focused on socioeconomic factors and they established an association between teenage pregnancy and factors such as low levels of education and low wealth quantile [15] initiation ceremonies [23] high school performance [24] early sexual debut and marriage [18]. Considering the multifactorial nature of determinants of teenage pregnancy, it is implausible to conclude that these are the only determinants of teenage pregnancy in Malawi. To the best of our knowledge, some factors that may influence teenage pregnancy such as use of contraception, domestic violence and the facility type and authority for the source of contraceptives have not been studied. Also notably, a potential source of selection or misclassification bias [25–27] exists in all studies conducted in Malawi by studying a population (girls aged 15–19 years) which was partially exposed to variables of interest.

It is against this gap that this study was conducted to identify factors that are associated with teenage pregnancies in Malawi through a community-based case-control study using country-representative secondary data from 2015 to 16 MDHS. The findings from this study will expand the knowledge of factors associated with teenage pregnancy in Malawi. Such knowledge will guide policymakers to develop focused and high-impact interventions to address the problem of teenage pregnancies and their effects in Malawi.

## Methods

### Study design

This was a community-based case-control study that used country-representative secondary data from the 2015-16 MDHS.

### Study setting

**Fig. 1:**
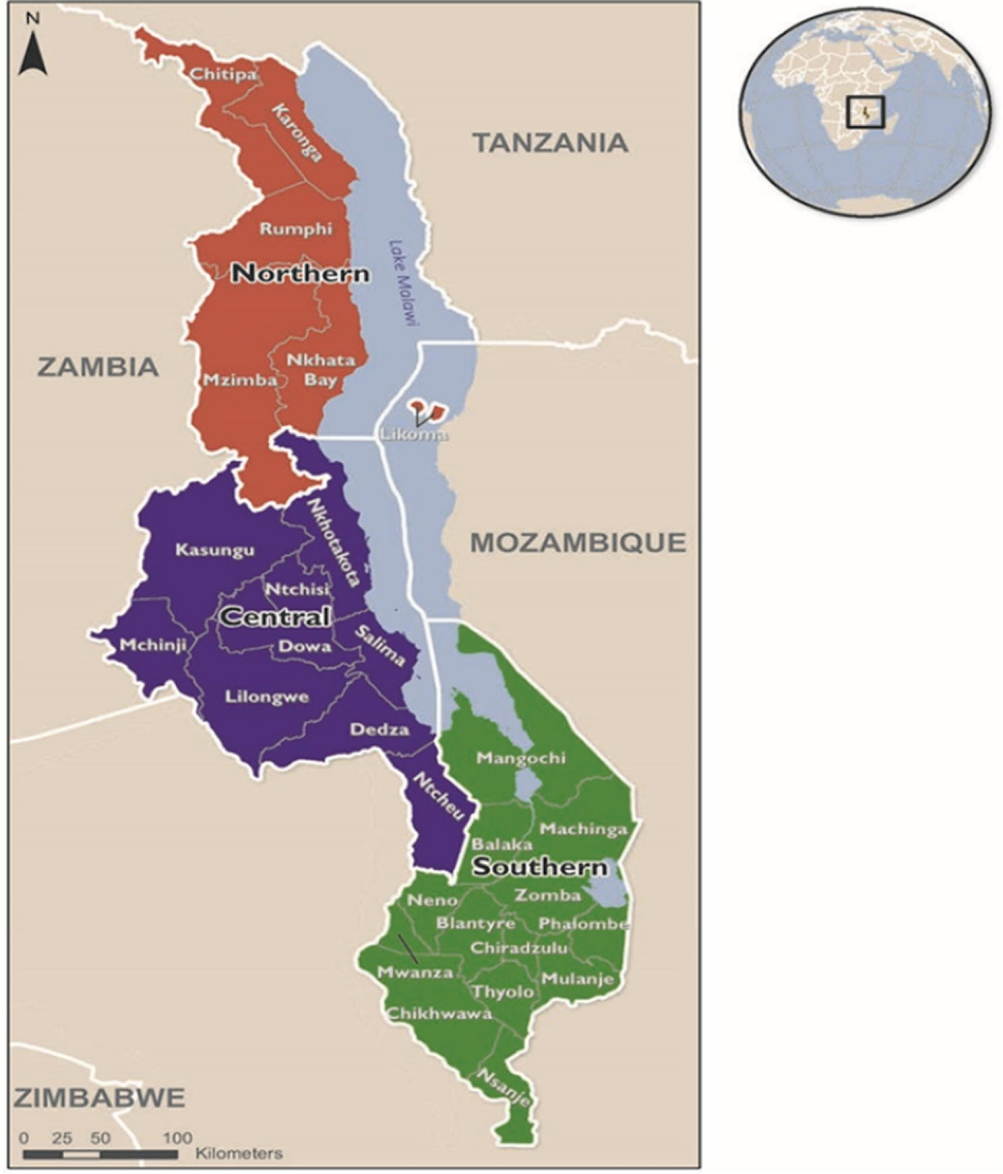
Map of Malawi. A square box inside the top right circle shows the location of Malawi in Africa. (Source: Malawi Service Provision Assessment 2013-14)

The study used secondary data from the 2015-16 MDHS collected from all 28 districts of Malawi (Fig. 1). Malawi is located in southeast Africa, latitude 13° 30’ S and longitude 34° 00’ E, with a land area of almost 119,000 square kilometres. The projected total population for 2021 is 19.6 billion [28], with youth aged 10–24 constituting more than one-third of the entire population [17]. The prevalence of teenage pregnancy in Malawi is 29% [15].

### Study Population

Women aged 20–24 from all 28 districts of Malawi who were surveyed in 2015-16 MDHS comprised the study population. The 20–24 age group was chosen to minimize misclassification bias [27] observed in previous studies whereby factors were studied in participants less than 20 years old, a population whose majority is partially exposed and still at risk.

### Study period

This study ran from September 2021 to October 2022.

### Sample size

**Fig. 2:**
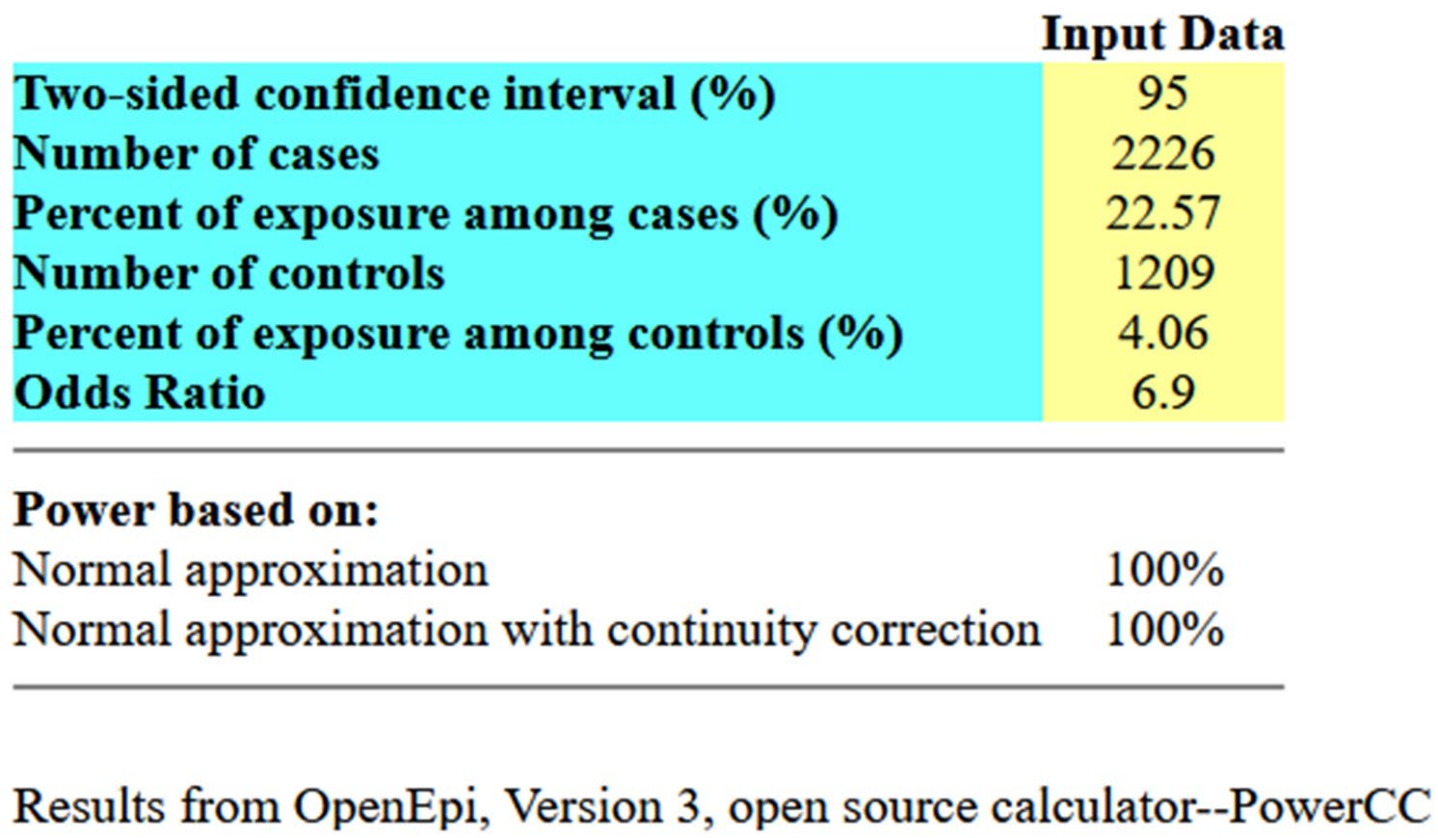
Input data for the case-control study power calculation

Since data were readily available, the sample size comprised all respondents who had completed questionnaires and residents of the same geographical location for a period of 5 years preceding the survey. This gave a total of 3,435 participants with more cases (respondents with a history of teenage pregnancy) than controls (respondents without a history of teenage pregnancy) at a ratio of 2:1. The 5 or more years period of residence was chosen to obtain a homogenous population with full exposure to the factors under study. Using the OpenEpi version 3 open-source calculator, this existing sample had 100% power to detect an odds ratio of 6.9 at a P value of 0.05 for “use of contraceptives,” assuming 4.06% prevalence in the controls and 22.57% prevalence in cases. The prevalence estimate for “contraceptive use” was obtained from a case-control study on determinants of teenage pregnancy conducted in Ethiopia [29]. The literature search did not yield any study in Malawi that collected prevalence data between cases and controls on determinants of teenage pregnancy, hence the use of the Ethiopian study. Figure 2 below shows input and output data for the power of the study calculation.

### Overview of the 2015-16 MDHS and the sampling method used

The MDHS are quantitative cross-sectional studies that are nationally representative and conducted in 5-year intervals with the primary objective of providing current estimates of basic demographic and health indicators [15]. The 2015-16 MDHS used a two-stage cluster design sampling method that included selecting enumeration areas as the first stage and choosing households as the second stage. All women aged 15–49 who were either permanent residents of the selected households or visitors who stayed in the home the night before the survey were eligible to be interviewed. The survey, which had a response rate of 99%, included 26,361 homes and 24,562 female respondents, of whom 5,094 were women aged 20–24 years. Informed oral consent was obtained from each respondent, and a parent or guardian provided consent for an adolescent under 18 years.

### Data collection

Datasets of different surveys conducted by the Demographics and Health Survey (DHS) program are made available to researchers who intend to use secondary data upon request from the program’s website. For this study, 2015-16 MDHS datasets in Stata format were requested and downloaded from the DHS website. Stata 16 software extracted the raw dataset from the Woman’s questionnaire. The Standard recode manual for DHS-7 [30], a Guide to DHS Statistics DHS-7 version 2 [31], and the 2015-16 MDHS Woman’s Questionnaire Do-File were used to develop a dataset variable identification tool. This tool was used to identify variables of interest from the dataset, and Stata 16 software extracted the identified variables.

### Study variables

Table 1 describes all the variables used in this study.

**Table 1:** Description of outcome and exposure variables

Variable name	Description
a) Outcome variable	
Teenage pregnancy	A binary variable in which 1 = first childbirth after 19 years and 2 = first childbirth before 20 years (teenage pregnancy).
**b) Exposure variables**	
Age at first marriage	A continuous variable indicating the age at first marriage of the respondent in years. This variable was used to generate Marital status variable.
Marital status	A categorical variable generated from Age at first marriage variable in which 1 = no teenage marriage, 2 = marriage at < 18 years, 3 = marriage at 18–19 years.
Education status of woman	A categorical variable of the highest level of education that the respondent ever attained in which 1 = no education, 2 = primary education, 3 = secondary education and 4 = any post-secondary education.
Exposure to media	A binary variable generated by collapsing three categories in MDHS dataset into two in which 0 = no exposure and 1 = exposure to one, two or all three types of media.
Wealth Index	Categorical variable in which 1 = poor, 2 = poorer, 3 = middle, 4 = richer and 5 = richest.
Religion	Categorical variable generated by collapsing eight variables in MDHS dataset into four in which 1 = Catholic, 2 = other Christians, 3 = Muslims and 4 = Other.
Knowledge of contraception	A binary variable generated by collapsing four categories in MDHS dataset in which 1 = knows contraception and 0 = does not know contraception.
Use of contraception	A binary variable generated by merging four categories in MDHS dataset into two in which 0 = User, 1 = Non-user.
Domestic violence by father or mother	A binary variable generated by merging two variables in the MDHS dataset, violence by mother and violence by father, in which 1 = Yes and 2 = No.
Place of residence	Binary variable in which 1 = urban and 2 = rural.
Facility type and authority for source of contraceptive services	A categorical variable generated by collapsing 24 categories for the last source of contraceptive service for current users in the MDHS dataset into five categories in which 1 = Government hospital, 2 = Government health center, 3 = CHAM hospital, 4 = CHAM health center and 5 = other.

### Data analysis

Using Stata 16, the data were analyzed in a “survey design” setting and weighted on the cluster level. All statistical commands were prefixed with the survey command “syv” to adjust the results of the command for the survey setting. The first step in the analysis was the calculation of frequencies and percentages for descriptive statistics. Logistic regression analyses were used to determine the factors. In univariable logistic regression analysis, Crude Odds Ratios (COR), 95% Confidence Intervals (CIs) and P values were calculated, and statistical significance was obtained at a P value < 0.1. Only factors that were statistically significant in univariable analysis were included in multivariable logistic regression, where Adjusted Odds Ratios (AORs), 95% CIs and P values were calculated to identify the most significant factors. In multivariable analyses, statistical significance was obtained at a P value < 0.05.

### Ethical considerations

Ethical clearance with approval number P.01/22/3541 was secured from the College of Medicine and Research Ethics Committee (COMREC), and permission to use 2015-16 MDHS datasets was obtained from the DHS program. All terms of use for DHS datasets were adhered to.

## Results

### Demographics and background characteristics of study participants

Data on 3435 women aged 20–24, of whom 2226 were cases and 1209 were controls, were analyzed. The demographics and background characteristics of the study participants are presented in Table 2. The majority of study participants (85.6%) got married in their teenage hood and attained primary education (62.3%). A higher proportion of cases and controls, 96.1% and 82.2%, respectively, were not exposed to media. The poorest category of the wealth index comprised the majority of study participants (25.0%), while in terms of religion, the higher proportion of participants belonged to Other Christian denominations (62.2%) other than Catholics. The majority of both cases and controls reported being aware of contraception (99.3%), but in terms of use, more cases were users (57.1%) than controls (46.1%). Very few cases (0.7%) and controls (1.0%) were subjected to domestic violence either by the father or mother. For both cases and controls, rural residents (85.9%) outnumbered urban residents. In terms of facility type and authority for source of contraceptive services, the majority of participants utilized Government health centers (58.2%) more than other available sources.

**Table 2:** Demographics and background characteristics of study participants (*N* = 3,435)

Variables	Teenage pregnancy				Total	(*N*%)	*P*- value
	Cases	(*n*%)	Controls	(*n*%)			
Marital status							
No teenage marriage	119	(5.2)	381	(31.7)	500	(14.4)	< 0.001
Teenage marriage (before 19 years)	2 107	(94.85)	828	(68.31)	2 935	(85.6)	
**Education status of women**							
No education	186	(9.3)	45	(4.4)	231	(7.6)	< 0.001
Primary education	1650	(75.1)	448	(38.5)	2098	(62.3)	
Secondary education	384	(15.2)	630	(48.8)	1014	(26.9)	
Higher	6	(0.4)	86	(8.3)	92	(3.2)	
**Exposure to media (newspaper, radio, TV)**							
Not exposed	2134	(96.1)	1002	(86.2)	3136	(92.6)	< 0.001
Exposed	92	(3.9)	207	(13.8)	299	(7.4)	
**Wealth Index**							
Poorest	598	(28.8)	195	(18.0)	793	(25.0)	< 0.001
Poorer	584	(26.6)	185	(16.5)	769	(23.1)	
Middle	443	(19.7)	200	(17.9)	643	(19.0)	
Richer	356	(15.2)	225	(16.5)	581	(15.6)	
Richest	245	(9.8)	404	(31.2)	649	(17.3)	
**Religion**							
Catholic	317	(14.4)	224	(19.2)	541	(16.0)	< 0.001
Other Christians	1548	(67.0)	841	(67.6)	2389	(67.2)	
Muslims	352	(18.1)	139	(13.0)	491	(16.3)	
Other	9	(0.5)	5	(0.3)	14	(0.4)	
**Knowledge of contraception**							
Knows contraception	2220	(99.6)	1196	(98.8)	3416	(99.3)	0.076
Does not know	6	(0.4)	13	(1.2)	19	(0.7)	
**Use of contraception**							
User	1267	(57.1)	321	(25.6)	1588	(46.1)	< 0.001
Non-user	959	(42.9)	888	(74.4)	1847	(53.9)	
**Domestic violence by father or mother**							
Yes	13	(0.5)	16	(1.0)	29	(0.7)	0.053
No	2213	(88.5)	1193	(99.0)	3406	(99.3)	
**Place of residence**							
Rural	1973	(90.6)	905	(77.2)	2878	(85.9)	< 0.001
Urban	253	(9.4)	304	(22.8)	557	(14.1)	
**Facility type and authority for source of contraceptive services**							
Government Hospital	207	(11.6)	62	(12.0)	269	(11.7)	< 0.001
Government Health center	707	(60.3)	138	(49.8)	845	(58.2)	
CHAM Hospital	38	(2.2)	7	(1.9)	45	(2.2)	
CHAM health center	41	(2.6)	6	(0.9)	47	(2.3)	
Other	274	(23.3)	108	(35.5)	382	(25.7)	
No access	959	(42.9)	888	(74.4)	1847	(58.9)	
N = Number of participants, n = frequency of participants, % = Percent, P value = Probability value							

### Univariable logistic regression analysis

In univariable logistic regression analysis, all variables were independently associated with teenage pregnancy at a 95% CI and P value < 0.1 (Table 3).

### Multivariable logistic regression analysis

In multivariable logistic regression (Table 3), those who had no teenage marriage were less likely (AOR 0.13, 95% CI 0.094–0.193, *p* < 0.001) to have teenage pregnancy than those who had a teenage marriage. The likelihood of teenage pregnancy was also lower in those who attained secondary education (AOR 0.26, 95% C1 0.198–0.338, *p* < 0.001) or higher education (AOR 0.039, 95% C1 0.0134–0.112, *p* < 0.001) than those who had primary education only. Being in the richest category of wealth index (AOR 0.51, 95% C1 0.329–0.811, *p* = 0.004) reduced the odds of teenage pregnancy by 0.51 times compared to those in the poorest category of wealth index. Regarding the use of contraception, the odds of teenage pregnancy were 3.08 times higher in users (AOR 3.08, 95% C1 2.10–4.52, *p* = 0.001) than in non-users. Those who were exposed to domestic violence by the father or mother were less likely to have teenage pregnancy (AOR 0.37, 95% CI 0.201–0.681, *p* = 0.001) than those who were exposed. Place of residence and Facility type and authority for source of contraceptive services were not statistically significant.

**Table 3:** Univariable and multivariable logistic regression analyses for factors associated with teenage pregnancy

Variable	Univariable logistic regression				Multivariable logistic regression		
	UOR	CI	*P*- value		AOR	CI	*P*- value
Marital status							
No teenage marriage	0.12	0.088–0.156	<0.001		0.13	0.094–0.193	<0.001
Teenage marriage (before 19 years)	REF				REF		
**Educational status of women**							
No education	1.09	0.744–1.585	0.669		1.12	0.740–1.702	0.587
Primary education	REF				REF		
Secondary education	0.16	0.130–0.197	< 0.001		0.26	0.198–0.340	< 0.001
Higher	0.02	0.009–0.663	< 0.001		0.039	0.013–0.112	< 0.001
**Exposure to media (newspaper, radio, TV)**							
Not exposed	REF				REF		
Exposed	0.26	0.182–0.359	< 0.001		0.73	0.407–1.31	0.291
**Wealth Index**							
Poorest	REF				REF		
Poorer	1.01	0.723–1.406	0.962		1.00	0.704–1.41	0.99
Middle	0.69	0.505–0.939	0.019		0.79	0.568–1.11	0.17
Richer	0.57	0.421–0.784	0.001		0.88	0.623–1.25	0.49
Richest	0.20	0.142–0.273	< 0.001		0.51	0.329–0.811	0.004
**Religion**							
Catholic	0.76	0.584–0.981	0.036		0.78	0.587–1.059	0.114
Other Christians	REF				REF		
Muslims	1.4	1.09–1.796	0.008		1.28	0.923–1.787	0.137
Other	1.98	0.546–7.164	0.299		0.94	0.30–2.95	0.916
**Knowledge of contraception**							
Knows contraception	REF				REF		
Does not know	0.33	0.092–1.194	0.091		0.42	0.115–1.546	0.193
**Use of contraception**							
User	3.86	3.15–4.74	< 0.001		3.08	2.10–4.52	< 0.001
Non-user	REF				REF		
**Domestic violence by father or mother**							
Yes	0.5	0.246–1.020	0.058		0.37	0.201– 0.681	0.001
No	REF				REF		
**Place of residence**							
Rural	REF				REF		
Urban	0.35	0.253–0.483	< 0.001		1.33	0.85–2.08	0.212
**Facility type and authority for source of contraceptive services**							
Government Hospital	3.73	2.47–5.64	< 0.001		1.12	0.612–2.05	0.712
Health center	4.68	3.54–6.18	< 0.001		1.28	0.837–1.95	0.257
CHAM Hospital	4.66	1.98–10.96	< 0.001		1.48	0.696–3.17	0.307
CHAM health center	11.34	3.85–33.38	< 0.001		2.4	0.956–6.029	0.062
Other types	2.54	1.90–3.38	< 0.001		*	*	*
No access	REF				REF		

UOR = Unadjusted Odds Ratio, AOR = Adjusted Odds Ratio, CI = Confidence Interval, P value = Probability value, REF = Reference Category, *Category omitted in the multivariable analyses because of collinearity

## Discussion

This study sought to identify factors associated with teenage pregnancy in Malawi. In the subsequent paragraphs, key study findings will be discussed.

After adjusting for the effect of all independent variables in multivariable analyses, the results showed that no teenage marriage, attaining secondary or higher education, and being in the highest wealth index category reduced the odds of teenage pregnancy (Table 3). These findings are broadly in agreement with previous studies conducted in Malawi [15, 18, 23, 24], Ethiopia [12, 32, 33], Nepal [34], Uganda [35] and other African countries [19, 20, 36, 37]. Not being married during teenagehood, higher education and a higher wealth index are highly interlinked factors, and their protective effect is well explained by existing literature. No teenage marriage reduces episodes of sexual encounters, which often result in teenage pregnancy [23]. Being in school keeps adolescent girls preoccupied, reduces chances for sexual activity and keeps them well informed of pregnancy prevention methods. Conversely, being out of school predisposes adolescent girls to risky sexual behaviour, early marriage and ultimately pregnancy [19, 20, 38]. Higher wealth status nullifies the need for early marriage for economic gains and offers an opportunity for continuing education [23, 38]. Global partnerships such as “Girls not Brides” [39] and a girl child initiative dubbed “keeping girls in school” implemented by the government of Malawi and its partners such as Save the Children International (SCI) [40] have been influenced by this body of scientific knowledge.

Use of contraceptives was another determinant revealed by this study. Contraceptive use increased the likelihood of teenage pregnancy among users (Table 3). Although this was a novel finding in the Malawian context, an analysis of the Philippines DHS on adolescent pregnancy [41] had the same finding. In this current study, lack of protective effect of contraceptive use can be explained in two ways. Firstly, it could be a result of reverse causality in the way that participants started using contraceptives after getting pregnant or giving their first birth. The 2015-16 MDHS substantiates this claim by showing that contraceptive use increased from 5% in women with zero parity to 60% in women who started bearing children [15]. Adding to this explanation is another study on determinants of contraceptive use conducted in Lilongwe, Malawi, in which youths with a living child were more likely to use contraceptives than those with no child or who had never given birth before [42]. Secondly, contraceptive use appeared to be a risk factor in this study due to incorrect or inconsistent use as demonstrated in a sub-Saharan Africa systematic review on predictors of teenage pregnancy [19]. On the other hand, case-control studies conducted in Ethiopia [29] and Uganda [35] showed that contraceptive use reduced the likelihood of teenage pregnancy among users. This contradictory finding may be due to differences in the study populations used. This current study recruited 20-24-year-old participants to minimize misclassification bias which could arise due to partial exposure to variables under investigation. The case-control studies in Ethiopia and Uganda recruited teenagers. It is possible that some of the younger adolescents who were classified as controls ended up being cases and contraceptive users later in their teenage hood. This is why this study opted to recruit women aged 20–24 to account for full exposure to the explanatory variables under study.

Domestic violence by the father or mother was found to be a protective factor (Table 3). This contradicts findings from a meta-analysis study dominated by studies conducted in Western countries that reported that those who had teenage pregnancy were more likely to have been exposed to physical abuse [22]. This inconsistency can be well understood from the definition of domestic violence as used in this current study. According to the data source, domestic violence was defined as physical violence involving hitting, slapping, kicking, or any other form of physical violence that could end up hurting the participant [15, 30]. This definition has elements of an authoritarian (strict parenting) parenting style commonly practised in African countries to instil discipline in children [43]. On that note, it is most likely that what study participants reported as domestic violence was a form of authoritarian parenting style that is also known to have an obedient effect on children [44]. In this study, this effect of obedience explains why those exposed to domestic violence by their parents were less likely to become pregnant since parents disapprove of teenage pregnancy for unmarried adolescents. In support of this claim is a study that was conducted in Ghana which showed that adolescents who belonged to families with authoritarian parents (those imposing strict rules and regulations on their children) were less likely to have teenage pregnancy [45].

Much as it anticipated that place of residence may determine teenage pregnancy due to contextual factors such as access to information, services and differential socioeconomic factors, this study found no significant association between place of residence and teenage pregnancy. This finding is consistent with studies conducted in Ethiopia [12, 29], Uganda [35] and five East African countries, including Malawi [18, 20]. All these studies found no significant association between teenage pregnancy and place of residence.

Facility type and authority for source of contraceptive services is a factor of much interest considering that it has not been studied in Malawi according to the reviewed published literature. This study did not find a significant association between this factor and teenage pregnancy. A significant finding on this factor would have provided a proxy indicator of the quality of health service factors such as YFHS provided by different service providers and their effect on teenage pregnancy. Given the fact that previous studies in some countries of sub-Saharan Africa found that the source of YFHS determines teenage pregnancy due to issues of quality [19, 46], the non – significant finding reported in this current study must be interpreted with caution. In this study, only half of the study participants were assessed on this variable due to limitations of secondary data use. This limitation may explain the non-significant result. There is evidence of poor quality of YFHS [17] and family planning services [47] in other health facilities in Malawi, however, a knowledge gap exists as to whether accessing contraceptives from these sources determines teenage pregnancy. The use of primary data or other research designs such as DHS spatial analysis may provide more reliable results on the association between teenage pregnancy and contraceptive sources.

### Study strengths and limitations

The use of a case-control study design provided an excellent statistical approach to compare two study groups, cases and controls, in the event of multiple exposures with a single outcome. The design is also suitable for the preliminary investigation of suspected factors, and findings from this study may be used to justify longitudinal studies later. The study results are generalizable because the study used country-representative data. The use of multivariable analyses allowed the study to adjust for the effects of other variables. Observer bias was significantly minimized in this study because the primary data were not collected for the purpose of this study.

This study is subject to the following limitations: the information provided by the respondents was self-reported and subjected to recall bias or concealing some of the information deemed confidential by the participant; ascertainment of outcome status did not include pregnancies lost through abortion; the use of secondary data made it impossible to study other variables because the dataset did not provide sufficient information on such; and the study design could not establish the temporal relationship between exposure and outcome.

## Conclusion

This study sought to identify the determinants of teenage pregnancy in Malawi. After adjusting for the effect of other independent variables in multivariable analyses, no teenage marriage; secondary education; higher education; being in the highest wealth index category; use of contraception; and domestic violence by the father or mother were statistically significant. Findings from this study will guide policymakers to develop focused and high-impact interventions to address the problem of teenage pregnancies in Malawi.

### Recommendations

Drawing from the list of identified factors associated with teenage pregnancy in this study, the following actions are highly recommended: The government should ensure effective implementation of the amended marriage legislation of 18 years as the legal age of marriage for Malawi [48]; sustain and expand the initiatives that promote girls’ education; introduce programs to address the low socioeconomic status of vulnerable adolescent girls; and find strategies to increase contraceptive use among teenagers before becoming pregnant. To address the study’s limitations and inconclusive results, future research should employ robust research methodologies that can yield more reliable results in determining the association between teenage pregnancy and health service factors such as source of contraceptives.

## Data Availability

The data that support the findings of this study are available from the DHS program, but restrictions apply to the availability of these data, which were used under license for the current study and thus are not publicly available. Data are, however, available from the corresponding author upon reasonable request and with permission of the DHS program.

## Abbreviations

AOR: Adjusted Odds Ratio
BLM: Banja La Mtsogolo
CHAM: Christian Health Association of Malawi
CI: Confidence interval
COMREC: College of Medicine Research and Ethics Committee
COR: Crude odds ratio
DHS: Demographic and Health Survey
ICPD: International Conference on Population and Development
MDHS: Malawi Demographic and Health Surveys
SCI: Save the Children International
YFHS: Youth Friendly Health Services

## References

[1] World Health Organization. Adolescent pregnancy [Internet]. WHO discussion papers on adolescence. 2004 [cited 2020 Sep 1]. https://apps.who.int/iris/bitstream/handle/10665/42903/9241591455_eng.pdf;jsess.

[2] World Health Organization. Adolescent pregnancy [Internet]. World Health Organization; 2020 [cited 2020 Sep 1]. https://www.who.int/news-room/fact-sheets/detail/adolescent-pregnancy.

[3] Chandra-Mouli, Venkatrama Camacho AV, Michaud P-A. WHO guidelines on preventing early pregnancy and poor reproductive outcomes among adolescents in developing countries. J Adolesc Heal [Internet]. 2013;52(5):517–22. 10.1016/j.jadohealth.2013.03.002 23608717

[4] Early and unintended pregnancy & the education sector [Internet]. 2017.

[5] World Health Organization. Adolescent pregnancy evidence brief [Internet]. 2018.

[6] Burton J. Highest teen pregnancy rates worldwide [Internet]. World Atlas. 2017 [cited 2021 Aug 31]. https://www.worldatlas.com/articles/highest-teen-pregnancy-rates-worldwide.html.

[7] UNAIDS. Global standards for quality health-care services for adolescents: a guide to implement a standards-driven approach to improve the quality of health care services for adolescents [Internet]. World Health Organization. 2015. http://apps.who.int/iris/bitstream/10665/183935/1/9789241549332_vol1_eng.pdf.

[8] National Statistical Office (NSO) and, Macro ICF. Malawi Demographic and Health Survey 2010 [Internet]. Zomba, Malawi, and Calverton, Maryland, USA: NSO and ICF Macro; 2011. 50 p. http://www.nsomalawi.mw/index.php?option=com_content&view=article&id=175&Itemid=46

[9] Ganchimeg T, Ota E, Morisaki N, Laopaiboon M, Lumbiganon P, Zhang J (2014). Pregnancy and childbirth outcomes among adolescent mothers: a World Health Organization multicountry study. BJOG.

[10] Papri FS, Khanam Z, Ara S, Panna MB. Adolescent pregnancy: risk factors, outcome and prevention. Chattagram Maa-O-Shishu Hosp Med Coll J [Internet]. 2016;15(1):53–6. https://www.researchgate.net/publication/305393238_Adolescent_Pregnancy_Risk_Factors_Outcome_and_Prevention.

[11] Mandiwa C, Namondwe B, Makwinja A, Zamawe C. Factors associated with contraceptive use among young women in Malawi: analysis of the 2015–16 Malawi demographic and health survey data. BMC [Internet]. 2018;3(1):1–9. 10.1186/s40834-018-0065-x. 10.1186/s40834-018-0065-x PMC6146597 30250748

[12] Ayele BG, Gebregzabher TG, Hailu TT, Assefa A. Determinants of teenage pregnancy in Degua Tembien District, Tigray, Northern Ethiopia : a community-based case-control study. PLoS One [Internet]. 2018;1–15. 10.1371/journal.pone.0200898. 10.1371/journal.pone.0200898 PMC6059451 30044850

[13] Loaiza E, Liang M. Adolescent pregnancy: a review of the evidence [Internet]. New York: UNFPA; 2013. 15 p. https://gsdrc.org/document-library/adolescent-pregnancy-a-review-of-the-evidence/.

[14] Guttmacher Institute. Adolescent pregnancy and its outcomes across countries [Internet]. Guttmacher Institute. 2015. https://www.guttmacher.org/fact-sheet/adolescent-pregnancy-and-its-outcomes-across-countries.

[15] National Statistical Office (NSO) [Malawi] and ICF. Malawi Demographic and Health Survey 2015-16 [Internet]. National Statistics Office. 2015. http://dhsprogram.com/pubs/pdf/FR319/FR319.pdf.

[16] World Health Organization. Contraception in adolescence [Internet]. WHO discussion papers on adolescence. 2004. http://apps.who.int/iris/bitstream/handle/10665/42901/9241591447_eng.pdf;jsessionid=57D75735F14E197A7AB2C57493BBBF93?sequence=1.

[17] Government of Malawi. Evaluation of Youth-Friendly Health Services in Malawi [Internet]. African Population and Health Research Center Management Sciences for Health, and PATH. 2014. www.e2aproject.org.

[18] Chirwa G, Mazalale J, Likupe G, Nkhoma D, Chiwaula L, Chitsanya J. An evolution of socioeconomic related inequality in teenage pregnancy and childbearing in Malawi. PLoS One [Internet]. 2019;14(11):1–16. https://journals.plos.org/plosone/article?id=10.1371/journal.pone.0225374. 10.1371/journal.pone.0225374 PMC6867649 31747437

[19] Gunawardena N, Fantaye AW, Yaya S. Predictors of pregnancy among young people in sub-Saharan Africa: a systematic review and narrative synthesis. BMJ Glob Heal [Internet]. 2019;4(3):1–8. https://pubmed.ncbi.nlm.nih.gov/31263589/. 10.1136/bmjgh-2019-001499 PMC6570986 31263589

[20] Wado YD, Sully EA, Mumah JN (2019). Pregnancy and early motherhood among adolescents in five east African countries: a multi-level analysis of risk and protective factors. BMC Pregnancy Childbirth.

[21] Romero L, Pazol K, Warner L, Cox S, Kroelinger C, Besera G et al. Reduced disparities in birth rates among teens aged 15–19 Years - United States, 2006–2007 and 2013–2014. MMWR Morb Mortal Wkly Rep [Internet]. 2016;65(16):2–8, 45–89. https://www.cdc.gov/mmwr/volumes/65/wr/pdfs/mm6516a1.pdf. 10.15585/mmwr.mm6516a1 27124706

[22] Madigan S, Wade M, Tarabulsy G, Jenkins JM, Shouldice M. Association between abuse history and adolescent pregnancy: a meta-analysis. J Adolesc Heal [Internet]. 2014;55(2):151–9. https://www.academia.edu/12759562/Association_Between_Abuse_History_and_Adolescent_Pregnancy_A_Meta_analysis. 10.1016/j.jadohealth.2014.05.002 25049043

[23] Munthali AC, Kok MC. Gaining insight into the magnitude of and factors influencing child marriage and teenage pregnancy and their consequences in Malawi [Internet]. 2016. https://www.kit.nl/wp-content/uploads/2018/10/Baseline-report-Malawi-Yes-I-Do.pdf.

[24] Glynn JR, Sunny BS, DeStavola B, Dube A, Chihana M, Price AJ (2018). Early school failure predicts teenage pregnancy and marriage: a large population-based cohort study in northern Malawi. PLoS ONE.

[25] Alexander LK, Lopes B, Ricchetti-Masterson K, Yeatts KB. Selection bias. ERIC Noteb [Internet]. Second Edi. https://sph.unc.edu/epid/eric/.

[26] Pham A, Cummings M, Lindeman C, Drummond N, Williamson T. Recognizing misclassification bias in research and medical practice. Fam Pract [Internet]. 2019;36(6):804–7. https://academic.oup.com/fampra/article/36/6/804/5628074. 10.1093/fampra/cmy130 31738429

[27] Spencer EA, Mahtani KR, Brassey JHC. Misclassification bias [Internet]. Catalogue Of Bias; 2018 [cited 2022 Sep 19]. https://catalogofbias.org/biases/misclassification-bias/.

[28] World Population Review. Malawi population 2021 [Internet]. World Population Review; 2021 [cited 2021 Sep 11]. https://worldpopulationreview.com/countries/malawi-population.

[29] Geda YF. Determinants of teenage pregnancy in Ethiopia: a case–control study, 2019. Curr Med Issues [Internet]. 2021;17(19):185–7. https://stacks.cdc.gov/view/cdc/87312.

[30] DHS Program/ICF. Standard recode manual for DHS-7 [Internet]. USAID. 2018. https://dhsprogram.com/pubs/pdf/DHSG4/Recode7_DHS_10Sep2018_DHSG4.pdf.

[31] Croft TN, Marshall AMJ, Allen CK. Guide to DHS statistics [Internet]. ICF., Rockville, Maryland USA. 2018. https://www.dhsprogram.com/pubs/pdf/DHSG1/Guide_to_DHS_Statistics_DHS-7_v2.pdf.

[32] Birhanu BE, Kebede DL, Kahsay AB, Belachew AB. Predictors of teenage pregnancy in Ethiopia: a multilevel analysis. BMC Public Health [Internet]. 2019;1–10. https://www.ncbi.nlm.nih.gov/pmc/articles/PMC6525551/. 10.1186/s12889-019-6845-7 PMC6525551 31101101

[33] Tigabu S, Liyew AM, Geremew BM. Modeling spatial determinates of teenage pregnancy in Ethiopia; geographically weighted regression. BMC Womens Health [Internet]. 2021;21(1):1–12. 10.1186/s12905-021-01400-7. 10.1186/s12905-021-01400-7 PMC8223368 34167542

[34] Poudel S, Upadhaya N, Khatri RB, Ghimire PR. Trends and factors associated with pregnancies among adolescent women in Nepal: pooled analysis of Nepal demographic and health surveys (2006, 2011 and 2016). PLoS One [Internet]. 2018;13(8):1–13. 10.1371/journal.%0Apone.0202107. 10.1371/journal.pone.0202107 PMC6084961 30092087

[35] Ochen AM, Chi PC, Lawoko S. Predictors of teenage pregnancy among girls aged 13–19 years in Uganda: a community based case-control study. BMC Pregnancy Childbirth [Internet]. 2021;231–231. 10.1186/s12884-019-2347-y. 10.1186/s12884-019-2347-y PMC6591948 31234816

[36] Kassa GM, Arowojolu AO, Odukogbe AA, Yalew AW (2018). Prevalence and determinants of adolescent pregnancy in Africa: a systematic review and meta-analysis. BMC Reprod Heal.

[37] Eyeberu A, Getachew T, Sertsu A, Sisay M, Baye Y, Debella A et al. Teenage pregnancy and its predictors in Africa: a systematic review and meta-analysis. Int J Health Sci (Qassim) [Internet]. 2022;16(6):47–60. PMC9682880 36475034

[38] Hunt F. Dropping out from school: a cross country review of literature [Internet]. 2008. http://www.create-rpc.org/pdf_documents/PTA16.pdf.

[39] Girls Not Brides. Our partnership [Internet]. Girls Not Brides; 2023 [cited 2023 May 23]. https://www.girlsnotbrides.org/our-partnership/.

[40] Save the Children. Project brief: keeping girls in school [Internet]. Save the Children. 2021. 1–2 p. https://resourcecentre.savethechildren.net/document/keeping-girls-school-cash-plus-education-programme-malawi/.

[41] Habito CM, Vaughan C, Morgan A. Adolescent sexual initiation and pregnancy: what more can be learned through further analysis of the demographic and health surveys in the Philippines? BMC Public Health [Internet]. 2019;19(1):1–13. 10.1186/s12889-019-7451-4. 10.1186/s12889-019-7451-4 PMC6701073 31429733

[42] Maruwo GB, Felix W, Muula AS, Zonda K, Kachale F. Factors associated with long-acting and short-acting reversible contraceptives use among 10–24 years-old youths in Lilongwe, Malawi. Front Public Heal [Internet]. 2022; https://www.frontiersin.org/articles/10.3389/frph.2022.949458/full. 10.3389/frph.2022.949458 PMC9580680 36303663

[43] Akande O, African. parents, don’t provoke your children [Internet]. TGC Africa Edition; 2021 [cited 2022 Sep 11]. https://africa.thegospelcoalition.org/article/african-parents-dont-provoke-your-children/.

[44] Cherry K. Why parenting styles matter when raising children [Internet]. verywellmind; 2022 [cited 2022 Sep 11]. https://www.verywellmind.com/parenting-styles-2795072.

[45] Ahinkorah BO, Hagan Junior JE, Seidu AA, Mintah JK, Sambah F, Schack T et al. Examining pregnancy related socio-cultural factors among adolescent girls in the Komenda-Edina-Eguafo-Abrem Municipality in the Central Region of Ghana: a case-control study. Front Public Heal [Internet]. 2019;7(APR):1–9. https://pubmed.ncbi.nlm.nih.gov/31069207/. 10.3389/fpubh.2019.00093 PMC6491621 31069207

[46] Yakubu I, Salisu WJ. Determinants of adolescent pregnancy in sub-saharan Africa: a systematic review. Reprod Health. 2018;15(1). 10.1186/s12978-018-0460-4 PMC5787272 29374479

[47] Ministry of Health [Malawi] and ICF International. Malawi Service Provision Assessment (SPA) 2013-14 [Internet]. 2013. https://dhsprogram.com/pubs/pdf/SPA20/SPA20%5BOct-7-2015%5D.pdf.

[48] UN Women. Malawi Parliament adopts amendment to end child marriage [Internet]. UN Women; 2017 [cited 2022 Sep 16]. https://www.unwomen.org/en/news/stories/2017/2/news-malawi-parliament-adopts-amendment-to-end-child-marriage.

